# The complete mitochondrial genome of the North American caddisfly *Anabolia bimaculata* (Insecta: Trichoptera: Limnephilidae)

**DOI:** 10.1080/23802359.2017.1372728

**Published:** 2017-09-01

**Authors:** Daniel S. J. Peirson, Jeffrey M. Marcus

**Affiliations:** Department of Biological Sciences, University of Manitoba, Winnipeg, Canada

**Keywords:** Illumina sequencing, mitogenomics, trichoptera, limnephiloidea, Limnephilidae

## Abstract

The caddisfly *Anabolia bimaculata*, is a rapidly-maturing caddisfly found in temporary ponds across western North America. Whole genome Illumina sequencing facilitated the assembly of a complete circular mitochondrial genome of 15,048 bp from *A. bimaculata* consisting of 78.2% AT nucleotides, 22 tRNAs, 13 protein-coding genes, two rRNAs and a control region arranged in the canonical and ancestral gene order found in insects. This gene order is consistent with the gene order found in most other caddisfly species. Maximum likelihood phylogenetic reconstruction places *A. bimaculata* within a monophyletic Order Trichoptera and sister to the primitive lepidopteran *Micropterix calthella* (Micropterigidae).

Aquatic caddisfly larvae (Insect Order Trichoptera) are excellent habitat quality bioindicators (Wissinger et al. 2003), but can be challenging to identify, so molecular tools for taxonomic identification are valuable (Ruiter et al. 2013). Here we report the first complete mitogenome for a New World caddisfly.

On 17–18 July 2015, a USDA blacklight trap (Winter 2000) was deployed to collect night-flying insects at the Living Prairie Museum (GPS 49.889607 N, −97.270487 W), 12.9 ha of relict prairie habitat in Winnipeg, Manitoba, Canada (Living Prairie Mitogenomics Consortium 2017). One adult *Anabolia bimaculata* (Limnephilidae), a rapidly-maturing caddisfly found in temporary ponds across western North America (Berté and Pritchard 1986), was trapped (specimen number 2015.07.17.018). The specimen was pinned and deposited in the Wallis Roughley Museum of Entomology, University of Manitoba (voucher JBWM0362999).

DNA was prepared (McCullagh and Marcus 2015) and sequenced by Illumina MiSeq (San Diego, CA) (Peters and Marcus 2017). The complete mitogenome for *A. bimaculata* (Genbank MF680449) was assembled by Geneious 10.1.2 from 8,292,766 paired 75 bp reads (total 0.63 Gb) using a *Eubasilissa regina* (Trichoptera: Phryganeidae) reference mitogenome (NC_023374.1) (Wang et al. 2014). Annotation was performed with reference to *E. regina* and *Hydropsyche pellucidula* (Trichoptera: Hydropsychidae, KT876876.1) mitogenomes (Linard et al. 2017). The complete *A. bimaculata* nuclear rRNA repeat (Genbank MF680448) was also assembled using a *Stenopsyche marmorata* reference sequence (Trichoptera: Stenopsychidae, LC094265.1) and annotated with respect to *S. marmorata* and *Attacus ricini* (Lepidoptera: Saturniidae, AF463459) repeats (Wang et al. 2003).

The circular 15,048 bp mitogenome assembly of *A. bimaculata* was made from 40,856 paired reads with nucleotide composition of 39.1% A, 14.1% C, 8.5% G, and 38.1% T. *Anabolia bimaculata* maintains complete synteny with nearly all other sequenced trichopteran mitogenomes and shows the canonical insect mitochondrial gene order. It lacks the rearrangements previously reported in the *H. pellucidula* mitogenome (Linard et al. 2017). *Anabolia bimaculata COX1* features an aberrant start codon (CGA) and three mitochondrial protein-coding genes (*ATP8, NAD1 NAD4*) have single-nucleotide (T) stop codons that are completed by the post-transcriptional addition of 3′ A residues. *Anabolia bimaculata* tRNAs have standard cloverleaf secondary structures except for trnS (AGN) which has the dihydrouridine arm replaced by a loop as determined by analysis in Mfold (Zuker 2003). The rRNAs (789 bp 12S and 1354 bp 16s) are composed of 84.7% AT while the putative control region (276 bp) is 82.8% AT.

We reconstructed a phylogeny using 13 protein coding genes from mitogenomes of *A. bimaculata*, five other trichopteran species, and representatives from related holometabolous insect orders. DNA sequences from each gene were aligned in CLUSTAL Omega (Sievers et al. 2011), concatenated, and analysed by maximum likelihood (ML) and parsimony in PAUP* 4.0b8/4.0d78 (Swofford 2002) (Figure 1). ML phylogenetic analysis places *A. bimaculata* within a monophyletic Trichoptera, sister to the primitive lepidopteran *Micropterix calthella* (Micropterigidae). The Trichoptera + *Micropterix* clade is sister to the remaining Lepidoptera. This unconventional placement may be due to missing data in *Micropterix*. The arrangement of taxa within the Trichoptera is consistent with prior published reports (Linard et al. 2017).

**Figure 1.**
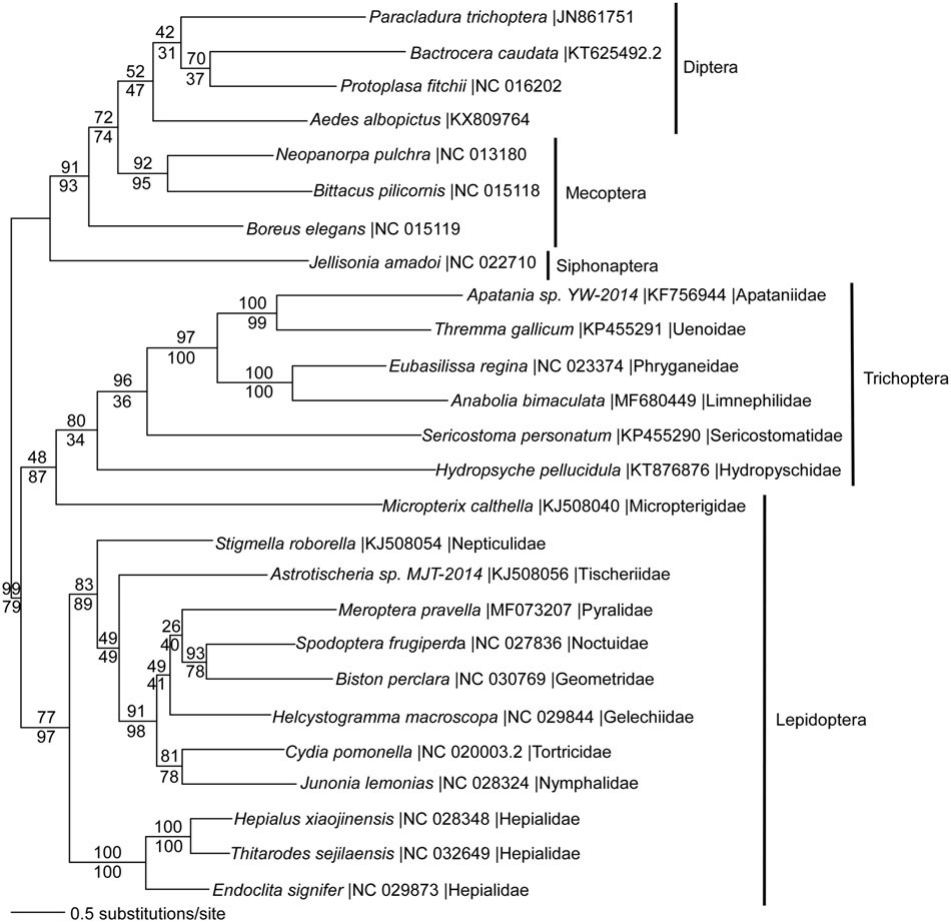
Maximum likelihood phylogeny (GTR + I + G model, I = 0.2790, G = 0.4760, likelihood score 153494.68624) of *Anabolia bimaculata* and other Trichoptera species with representatives from the sister-order Lepidoptera (moths and butterflies), and from Diptera (flies), Mecoptera (scorpionfiles), and Siphonaptera (fleas) based on 1 million random addition heuristic search replicates (with tree bisection and reconnection) of mitochondrial protein coding genes. One million maximum parsimony heuristic search replicates produced a single tree (37,008 steps) with nearly identical tree topology except that *Micropterix* is the sister taxon to *Hydropsyche*, rather than to the entire trichopteran clade. Numbers above each node are maximum likelihood bootstrap values and numbers below each node are maximum parsimony bootstrap values (each from 1 million random fast addition search replicates).
